# Non-Islet Cell Tumor Hypoglycemia Masquerading as Diabetes Remission in a Patient With Type 2 Diabetes Mellitus

**DOI:** 10.1210/jcemcr/luaf242

**Published:** 2025-10-30

**Authors:** Louisa Cheong, Pei Chia Eng, Andre Teck-Huat Tan, Wai Kit Cheong, Chin Meng Khoo, Doddabele Srinivasa Deepak

**Affiliations:** Division of Endocrinology, Department of Medicine, National University Hospital, Singapore 119228, Singapore; Division of Endocrinology, Department of Medicine, National University Hospital, Singapore 119228, Singapore; Department of Medicine, National University of Singapore, Singapore 117597, Singapore; Division of Endocrinology, Department of Medicine, National University Hospital, Singapore 119228, Singapore; Division of Endocrinology, Department of Medicine, Alexandra Hospital, Singapore 159964, Singapore; Division of Colorectal Surgery, Department of Surgery, National University Hospital, Singapore 119228, Singapore; Division of Endocrinology, Department of Medicine, National University Hospital, Singapore 119228, Singapore; Department of Medicine, National University of Singapore, Singapore 117597, Singapore; Division of Endocrinology, Department of Medicine, National University Hospital, Singapore 119228, Singapore

**Keywords:** diabetes remission, non-islet cell tumor hypoglycemia (NICTH), solitary fibrous tumor (SFT), insulin-like growth factor-2 (IGF-2)

## Abstract

Non-islet cell tumor hypoglycemia (NICTH) is a rare paraneoplastic phenomenon of tumor insulin-like growth factor (IGF)-2 hypersecretion. We report a case of a man with poorly controlled diabetes who experienced rapid glycemic improvement after 1 year of oral glucose-lowering therapy. He was diagnosed as achieving diabetes remission after medication cessation. However, he began experiencing recurrent symptomatic hypoglycemia after medication cessation. During a supervised fast, venous glucose dropped to 46.9 mg/dL (SI: 2.6 mmol/L) (reference range, 72.1-140.5 mg/dL [SI: 4.0-7.8 mmol/L]), with suppressed serum C-peptide and insulin, but undetectable beta-hydroxybutyrate levels. Venous glucose rose 37.8 mg/dL (SI: 2.1 mmol/L) (46.9 to 84.7 mg/dL [SI: 2.6 to 4.7 mmol/L]) following intravenous glucagon administration at fast cessation. Serum IGF-1 was low with raised IGF-2, and the IGF-2 to IGF-1 ratio was 33:1, confirming IGF-2 excess. Imaging revealed a large central-lower abdominal mass. He was started on prednisolone and frequent meals to prevent hypoglycemia before tumor resection. Histology revealed a solitary fibrous tumor. Postoperatively, his hypoglycemia resolved. NICTH can complicate glycemic assessment in concomitant diabetes. Clinicians should suspect underlying hypoglycemic disorders in persons with diabetes who develop unexpected glycemic improvement or hypoglycemic episodes despite medication reduction or cessation.

## Introduction

Non-islet cell tumors and insulinomas are rare tumors that typically present with spontaneous hypoglycemia. Clinical diagnosis of these conditions can be challenging, as hypoglycemic symptoms may be nonspecific and mimic other conditions. Diagnostic challenges arise when paraneoplastic hypoglycemia occurs in persons with diabetes, where hypoglycemic symptoms may be masked by preexisting insulin resistance or attributed to glucose-lowering medications, resulting in a delayed or missed diagnosis. We report a case of a pelvic solitary fibrous tumor (SFT) causing non-islet cell tumor hypoglycemia (NICTH) in a patient with type 2 diabetes mellitus, initially thought to be in diabetes remission, who eventually developed symptomatic hypoglycemic episodes.

## Case Presentation

A 50-year-old man had been diagnosed with type 2 diabetes 4 years previously. At diagnosis, his glycated hemoglobin A1c (HbA1c) was 12.9%. He was commenced on empagliflozin and metformin once daily by his primary care physician. His HbA1c improved to 5.9% within 4 months, and the medications were stopped less than 1 year after diagnosis. His HbA1c levels were maintained at < 6% without lifestyle changes. As such, he was deemed to have achieved remission from diabetes. He subsequently presented with a 6-month history of recurrent symptomatic hypoglycemic episodes. He reported tremors and light-headedness occurring more than 2 times per week with capillary blood glucose (CBG) readings as low as 48.7 mg/dL (SI: 2.7 mmol/L). The hypoglycemia episodes occurred predominantly at fasting and were exacerbated by exercise but resolved with consumption of sweets or food. Due to increased snacking and reduced exercise to circumvent hypoglycemia, he gained 15 kg in 6 months. Apart from the hypoglycemic episodes, he had been well with no intercurrent illnesses. He was no longer on any glucose-lowering medications and denied usage of traditional medications, over-the-counter supplements, or illicit drugs.

## Diagnostic Assessment

On examination, his body mass index was 33.7 kg/m^2^, and abdominal palpation revealed a hard, nontender suprapubic mass extending to the umbilicus. Blood counts, and renal, liver, and thyroid function tests were unremarkable. His 8 Am cortisol was adequate, and HbA1c was 5.2%. He underwent a 72-hour supervised fast, during which he developed hypoglycemia at 7 hours into the fast with a venous glucose of 46.9 mg/dL (SI: 2.6 mmol/L) (reference range, 72.1-140.5 mg/dL [SI: 4.0-7.8 mmol/L]). The corresponding serum C-peptide was low at 0.09 ng/mL (SI: 31 pmol/L) (reference range, 0.8-5.2 ng/mL [SI: 260-1729 pmol/L]) with a suppressed serum insulin < 1.6 mIU/L (SI: < 11.1 pmol/L) (reference range, 2.6-24.9 mIU/L [SI: 18.1-172.9 pmol/L]) and undetectable serum beta-hydroxybutyrate (BOHB) < 6.2 mg/dL (SI: < 0.6 mmol/L) (reference range, < 6.2 mg/dL [SI: < 0.6 mmol/L]). Venous glucose increased from 46.9 to 84.7 mg/dL (SI: 2.6 to 4.7 mmol/L) with intravenous 1 mg glucagon administered at fast cessation, suggesting an insulin-like effect (Table 1). Sulfonylurea screen and insulin antibodies were negative. Serum insulin-like growth factor (IGF)-1 and growth hormone (GH) levels were low with a significantly elevated IGF-2. The IGF-2 to IGF-1 ratio was 33:1, confirming IGF-2 excess (Table 2).

**Table 1.** Table showing supervised fast results

Time since fast initiation	Capillary blood glucose	Venous glucose	Serum beta-hydroxybutyrate	Serum C-peptide	Serum insulin
0 hours	93.7 mg/dL (SI: 5.2 mmo/L)				
4 hours	66.7 mg/dL (SI: 3.7 mmol/L)				
7 hours	50.4 mg/dL (SI: 2.8 mmol/L)	46.9 mg/dL (SI: 2.6 mmol/L)	<6.2 mg/dL (SI: <0.6 mmol/L)	0.093 ng/mL (SI: 31 pmol/L)	<1.6 mIU/L (SI: <11.1 pmol/L)

Intravenous glucagon 1 mg administered at fast termination					
Time since Glucagon given	Capillary blood glucose	Venous glucose	Serum beta-hydroxybutyrate	Serum C-peptide	Serum insulin
0 minutes		46.9 mg/dL (SI: 2.6 mmol/L)			
10 minutes		75.7 mg/dL (SI: 4.2 mmol/L)			
20 minutes		84.7 mg/dL (SI: 4.7 mmol/L)			
30 minutes		79.3 mg/dL (SI: 4.4 mmol/L)			

Hypoglycemia was precipitated at 7 hours into supervised fast with associated biochemical picture consistent with insulin-like effect of suppressed beta-hydroxybutyrate (BOHB) and low insulin and C-peptide levels, further confirmed by significant venous glucose rise from 46.9 to 84.7 mg/dL (SI: 2.6 to 4.7 mmol/L) with intravenous glucagon 1 mg administered at fast termination.

**Table 2.** Table showing growth hormone, IGF-1, and IGF-2 results, with calculated IGF-2 to IGF-1 ratio

Test name	Result	Reference range
Growth hormone	0.25 ng/mL (SI: 0.25 µg/L)	≤2.47 mg/mL (SI: ≤2.47 µg/L)
Insulin-like growth factor 1	34 ng/mL (SI: 4.4 nmol/L)	79-205 ng/mL (SI: 10.3-26.8 nmol/L)
Insulin-like growth factor 2	1154 ng/mL (SI: 150.9 nmol/L)	333-967 ng/mL (SI: 43.5-126.4 nmol/L)
IGF-2 to IGF-1 ratio	33	<3

Insulin growth factor 2 (IGF-2) levels were elevated with low IGF-1 levels, resulting in an elevated IGF-2 to IGF-1 ratio of 33:1, consistent with IGF-2 excess.

**Figure 1.**
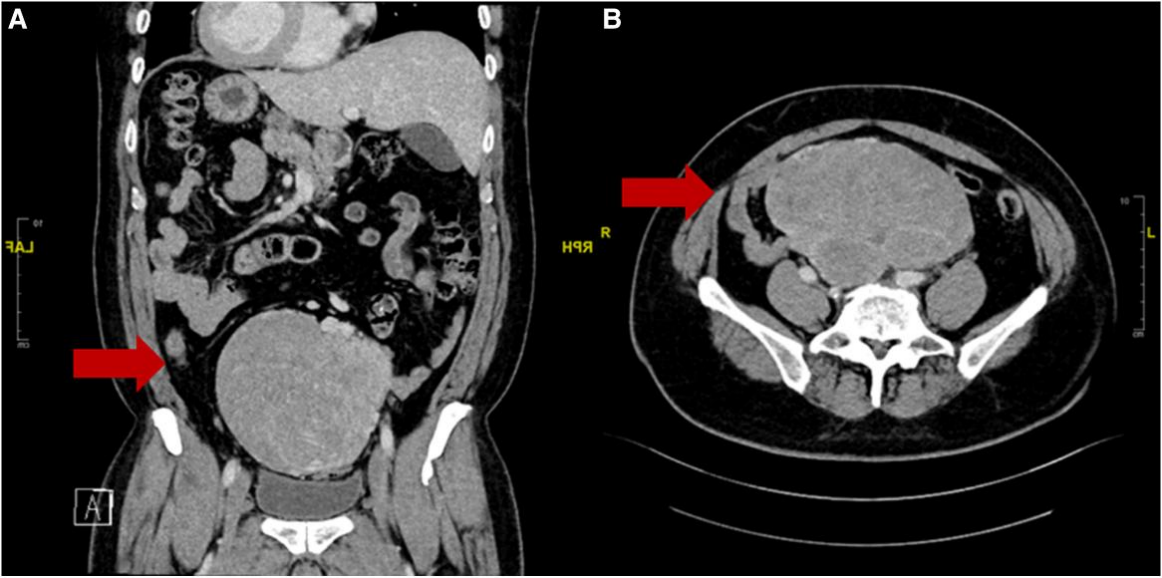
Computed tomography images of the abdomen and pelvis in the coronal (A) and transverse (B) planes demonstrating large heterogenous abdominal mass extending from suprapubic area to lower abdomen (arrows).

**Figure 2.**
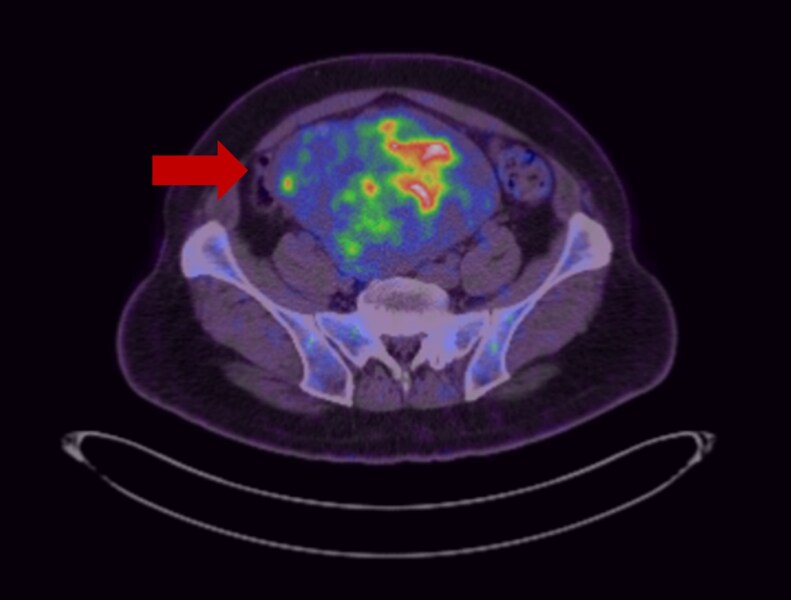
^18^F-Fluorodeoxyglucose positron emission tomography (FDG PET) image demonstrating the heterogenous FDG avidity in the lower abdominal mass (arrow).

Computed tomography of the abdomen and pelvis revealed a 15.5 × 11.5 × 13.4 cm lobulated mass in the central-lower abdomen and pelvis with areas of necrosis (Fig. 1). A ^18^F-fluorodeoxyglucose positron emission tomography (FDG PET) scan revealed a heterogeneously FDG-avid lobulated lower abdominopelvic mass with no other metabolically active metastases (Fig. 2). Imaging-guided biopsy revealed an atypical mesenchymal neoplasm with signal transducer and activator of transcription 6 (STAT6) expression, suggestive of a solitary fibrous tumor (SFT).

## Treatment

**Figure 3.**
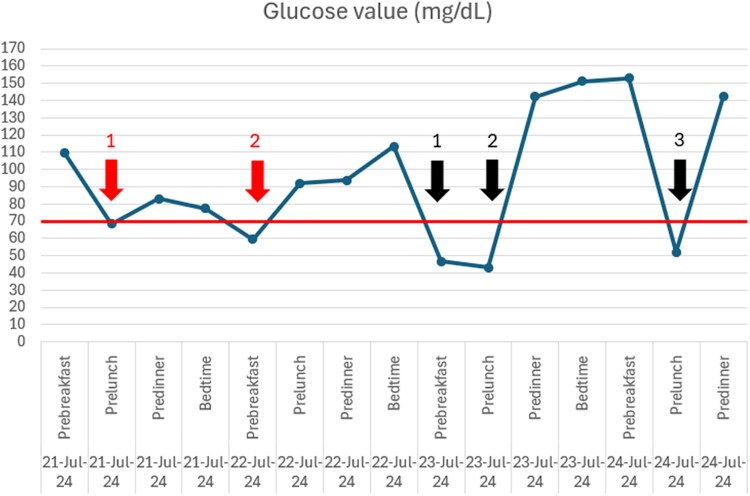
Image of the patient's inpatient capillary blood glucose (CBG) trends. When allowed to eat normally, the patient's CBG spontaneously decreased to 68.5 mg/dL (SI: 3.8 mmol/L) pre-lunch (red arrow 1) and 59.5 mg/dL (SI: 3.3 mmol/L) pre-breakfast (red arrow 2). When he was kept fasted for scans and procedures, his CBG down trended to 46.8 mg/dL (SI: 2.6 mmol/L) (black arrow 1), 43.2 mg/dL (SI: 2.4 mmol/L) (black arrow 2) and 52.2 mg/dL (SI: 2.9 mmol/L) (black arrow 3).

**Figure 4.**
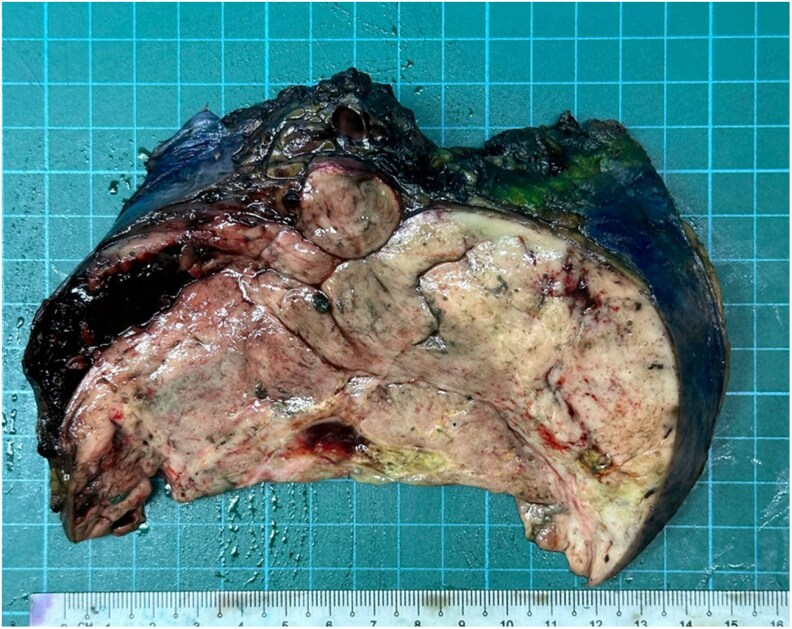
Image of the gross appearance of the patient's pelvic tumor, measuring 15 cm in its greatest dimension.

**Figure 5.**
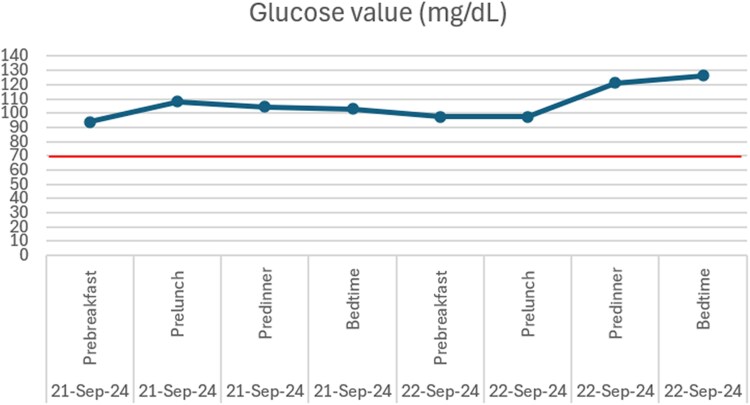
Image of the patient's postoperative capillary blood glucose trend demonstrating postoperative euglycemia without the need for dextrose-containing infusions or steroids.

A diagnosis of a pelvic SFT complicated by NICTH was made, and the patient was offered definitive surgical resection. While awaiting surgery, he experienced recurrent symptomatic hypoglycemia despite dietary modifications including frequent meals with complex carbohydrates (Fig. 3). He was commenced on prednisolone 20 mg every evening which resolved his hypoglycemic episodes. He subsequently underwent resection of the SFT, which measured 15 cm (Fig. 4) with no invasion into surrounding structures or gross liver and peritoneal disease. The patient made an uneventful postoperative recovery with no further hypoglycemia episodes (Fig. 5).

Histology revealed a spindle-cell neoplasm with STAT6 expression and molecular testing via RNA next-generation sequencing returned positive for *NGFI-A-binding protein 2 (NAB2)-STAT6* gene fusion favoring an SFT. Atypical features, including the lack of CD34 expression, were noted, portending a potentially malignant or dedifferentiated SFT with high-risk features for metastasis. He was further reviewed by Oncology and commenced on adjuvant chemotherapy.

## Outcome and Follow-Up

Although the patient has remained well postoperatively, a repeat postoperative IGF-2 measurement could not be obtained due to ongoing laboratory assay issues. He reports sustained euglycemia with no recurrence of hypoglycemic symptoms at 1 year after surgery. His HbA1c remains < 6% without diabetes treatment. He has been counseled on the potential for worsening glycemic control given the possibility that his previous diabetes was masked by concomitant NICTH.

## Discussion

NICTH is defined as a syndrome of hypoglycemia associated with neoplasms other than insulinomas [1]. It occurs due to paraneoplastic IGF-2 overproduction by the culprit tumor, which is often incompletely processed IGF-2 (big IGF-2) resulting from aberrant IGF-2 gene expression and precursor processing [1]. IGF-2 shares a high structural homology to insulin with actions at the IGF-1 receptor (IGF-1R), insulin receptor (IR) and hybrid IGF-1R/IR [2]. When IGF-2 secretion is increased, as in NICTH, increased binding of high circulating IGF-2 to IR can induce insulin-like effects, including hypoglycemia [3]. The effects of IGF-2 excess are potentiated by altered binding to IGF-binding proteins (IGFBP) and acid labile subunits (ALS). IGF-2 binding within complexes moderates its size, capillary permeability, and, thus, biological activity [4]. IGFBP-3 and ALS production are reduced due to negative feedback suppression of GH by IGF-2 excess [5]. Additionally, the structure of big IGF-2 has been shown to interfere with ALS binding [6]. Increased IGF-2 bioavailability [7] results in downstream insulin-like effects including peripheral glucose uptake by skeletal muscles and inhibition of lipolysis for gluconeogenesis [8]. Hypoglycemia is further exacerbated by IGF-2 binding to IGF-1R and IR at the pancreatic alpha cells and hypothalamus, which mimics the effects of insulin and IGF-1 to suppress secretion of counterregulatory hormones—glucagon and GH [9]. Additionally, tumor growth is enhanced by autocrine and paracrine effects of high local IGF-2 concentrations, which stimulates IGF-1R and IR expressed on tumor cells [10]. This results in a vicious cycle of tumor growth and worsening IGF-2 overproduction.

Similarly to insulinoma, NICTH typically presents with fasting hypoglycemia [11]. Although there is currently no consensus on diagnostic criteria, the use of the IGF-2 to IGF-1 ratio rather than absolute IGF-2 levels for diagnosis is recommended, as low GH and IGF-1 levels are expected [11] but IGF-2 levels may be normal depending on the proportion of IGF-2 precursors converted to mature IGF-2 for measurement by current available laboratory assays [12]. Elevated IGF-2 to IGF-1 ratios above 3:1 are suspicious for IGF-2 excess, with ratios > 10:1 virtually diagnostic for NICTH [11, 13].

NICTH was first described in 1929 by Nadler et al, who reported a case of hypoglycemia in a patient with hepatocellular carcinoma, initially attributed to impaired hepatic glucose mobilization [14]. It is typically associated with mesenchymal or epithelial tumors [15]. For our patient, histology of his pelvic mass revealed an SFT. SFTs are rare mesenchymal neoplasms that can arise from any location, most commonly from the pleura [16]. NICTH arising from SFTs is eponymously known as Doege-Potter syndrome, reported by Doege and Potter in 1930 as a case of hypoglycemia associated with a thoracic SFT [17]. This rare syndrome occurs in < 5% of SFT cases [16]. Peak incidence of SFTs occurs in the fifth to sixth decades of life, affecting both genders equally [18]. The molecular hallmark of SFTs is the presence of the *NAB2-STAT6* fusion gene on chromosome 12q13 [19], which was also detected in our patient's tumor. Although these tumors are typically benign, malignant potential and recurrence after surgical resection is associated with larger tumors, elevated mitotic activity, nuclear pleomorphism, tumor necrosis, and infiltrative borders [20].

Definitive management of NICTH involves surgical resection of the primary tumor [21]. Complete tumor resection is curative for hypoglycemia [22], with recurrence of hypoglycemia commonly signaling tumor recurrence [23]. There are currently no standardized guidelines on postoperative monitoring for NICTH. Postoperative IGF-1 and IGF-2 measurement for calculation of IGF-2 to IGF-1 ratios have been used to assess for biochemical cure in various case reports, but the timing of measurement varies widely from days to weeks postoperatively [24, 25].

Our patient presented with fasting hypoglycemia preceded by rapid improvement of his diabetes control allowing for cessation of treatment, which had been interpreted as diabetes remission despite weight gain and a lack of lifestyle changes. The insulin-like effect of IGF-2 hypersecretion from the enlarging tumor could have possibly contributed to his initial rapid glycemic improvement and apparent remission of diabetes. As the tumor size and associated IGF-2 excess increased further, overt symptomatic hypoglycemia then developed. This highlights the potential challenge in diagnosing paraneoplastic hypoglycemic disorders in individuals with concomitant diabetes, as hypoglycemia may be masked by preexisting insulin resistance or mistakenly attributed to factors such as diabetic medications.

Unexplained glycemic improvement has been linked to the diagnosis of insulinomas in individuals with diabetes in the literature. Nastos et al [26] described a case of a woman who experienced unexplained normalization of blood glucose levels despite cessation of diabetic medications, followed by hypoglycemic episodes, and was subsequently diagnosed with an insulinoma. Similarly, Singbo et al [27] reported a case of a man with apparent well-controlled diabetes, despite poor adherence to treatment, who developed recurrent hypoglycemic episodes and was discovered to have an insulinoma. Recurrent hypoglycemia in patients with diabetes despite cessation of diabetes medications leading to the diagnosis of insulinomas has also been reported [28, 29]. In contrast, the existing literature surrounding NICTH co-occurring with diabetes is limited. Despite case reports of recurrent hypoglycemia caused by NICTH, ours is the first to report a case of NICTH in an individual with diabetes resulting in apparent diabetes remission. A literature review of 76 published SFT-associated NICTH cases in 2017 by Han et al [30] found that SFTs resulting in NICTH were more likely to be malignant and extra-thoracic, and male patients more likely to present with hypoglycemia. However, there was no mention of concomitant diabetes in the cases included. Despite the lack of literature on NICTH co-existing with diabetes, similarities in the pathophysiology of hypoglycemia in NICTH and insulinomas leads to the potential for NICTH to present similarly in persons with diabetes.

## Learning Points

- Paraneoplastic hypoglycemic conditions, including NICTH, have the potential to develop in any patient, including persons with diabetes.
- Paraneoplastic hypoglycemia may mimic spontaneous diabetes remission in individuals with concomitant diabetes due to alterations in glucose metabolism.
- Clinicians should maintain a high index of suspicion for underlying hypoglycemic disorders in patients with diabetes who develop apparent remission or recurrent hypoglycemic episodes despite medication reduction or cessation.

## Contributors

All authors made individual contributions to authorship. L.C., A.T.H.T., and D.S.D. were involved in the diagnosis and management of the patient. W.K.C. was responsible for the patient's surgery. L.C., P.C.E., and C.M.K. were involved in the conceptualization and writing of the original draft of the manuscript. L.C., P.C.E., C.M.K., and D.S.D. were involved in editing of the manuscript. All authors reviewed and approved the final draft.

## Data Availability

Data sharing is not applicable to this article as no datasets were generated or analyzed during the current study.

## Abbreviations

ALS: acid labile subunit
FDG: fluorodeoxyglucose
GH: growth hormone
HbA1c: glycated hemoglobin
IGF: insulin-like growth factor
IGF-1R: insulin-like growth factor 1 receptor
IR: insulin receptor
NICTH: non-islet cell tumor hypoglycemia
PET: positron emission tomography
SFT: solitary fibrous tumor
STAT6: signal transducer and activator of transcription 6
